# Female Trans-Sphincteric Anterior Anal Fistula: Still an Unsolved Problem—Results from a Nationwide Cohort Study

**DOI:** 10.3390/jcm14207326

**Published:** 2025-10-16

**Authors:** Alba Correa Bonito, Óscar Cano Valderrama, Manuel Muinelo Lorenzo, Begoña Ochoa Villalabeitia, Juan Ocaña Jiménez, Beatriz Martín Pérez, Lidia Cristóbal Poch, Tamara Fernández Miguel, Carlos Cerdán Santacruz, on behalf of Young Group of Spanish Coloproctology Association

**Affiliations:** 1 Hospital Universitario de La Princesa, 28006 Madrid, Spain; alba.bonito90@gmail.com; 2 Complejo Hospitalario Universitario de Vigo, 36312 Vigo, Spain; oscarcanovalderrama@hotmail.com (Ó.C.V.); manuel.muinelo.lorenzo@sergas.es (M.M.L.); 3 Hospital Galdakao, 48960 Bizkaia, Spain; bego_ochoa@hotmail.com (B.O.V.); tamara.fdez.miguel@gmail.com (T.F.M.); 4 Hospital Universitario Ramón y Cajal, 28034 Madrid, Spain; jocajim@gmail.com; 5 Hospital Universitario de Badajoz, 06080 Badajoz, Spain; beatriz_martin1@yahoo.com; 6 Hospital Universitario Marqués de Valdecilla, 39008 Santander, Spain; lidiacristobalpoch@gmail.com

**Keywords:** female, anal anterior trans-sphincteric fistula, surgery, healing, recurrence, fecal incontinence

## Abstract

**Background/Objectives**: Female trans-sphincteric anterior anal fistula is one of the most challenging fistulae because of the inherent risk of postoperative incontinence. The objective of this study is to analyze current surgical practices in these patients and their results. **Methods**: This study is a nationwide cohort retrospective study of all female patients with trans-sphincteric anterior anal fistula that were operated on in 2019. The primary outcomes measured were the analysis of the different techniques used in this type of patient and the results in terms of healing, persistence and recurrence. **Results**: We analyzed 139 patients that were operated on in 2019 because of an anterior trans-sphincteric fistula. The most usual technique performed was fistulotomy (29.5%), followed by ligation of the inter-sphincteric fistula tract (22.3%). The overall healing rate was 60.4%, taking into account that this rate was higher for fistulotomy (87.8%) than for sphincter-preserving techniques such as ligation of the inter-sphincteric fistula tract, advanced flap and fistulotomy plus sphincterography (51.8%). The main protective factors for healing that have been identified are the absence of current smoking and the realization of a fistulotomy. The overall postoperative incontinence rate is 12.6%, which is higher in patients with higher fistula (25%). It is important to highlight that in low fistula, the rate of incontinence in techniques different from fistulotomy is about 25%. The only risk factor for incontinence that has been identified is obesity. **Conclusions**: The treatment of this type of fistula is still a big challenge with high diversity in terms of surgical techniques, even though, fistulotomy still remains safe and with good results in terms of healing and incontinence in low fistulas and medium fistulas with strict selection criteria. In these cases, fistulotomy is the indicated procedure.

## 1. Introduction

The treatment of anal fistulae (AF) is still nowadays a challenge for most surgeons, moreover if less favorable anatomies of AF are implied in groups of patients at risk of anal incontinence, such as female trans-sphincteric anterior anal fistula (F-TAAF).

In this group of patients, it is well known that the increase in the risk of fecal incontinence (FI) could be attributed to obstetric history [1] and to specific anatomic features of the anterior perineal region, such as weakness of the sphincteric apparatus at that level, the narrowness of the perineal body and the diminished length of the anal canal. Therefore, the need to not damage the sphincteric apparatus during potential surgeries for the treatment of anal fistulae is capital. To preserve the anal sphincter function in these patients, new sphincter-preservation procedures have been developed. However, the healing rate of these procedures in this type of patient has been scarcely studied. Therefore, studies analyzing F-TAAF are needed to determine whether these new procedures can be effectively used in the treatment of these patients.

We could say that F-TAAF, even though not anatomically complex because of the length and distribution of the tract [2], could indeed be named as such because of the features of the patients we are confronting. These fistulae would be complex because of the vulnerability of the sphincteric apparatus of the patients affected and the challenge that its treatment implies.

To date, there are no publications concerning the optimal treatment for this type of fistulae in these patients in terms of healing and fecal incontinence, for which the development of this study pretends to enlighten this so frequent matter. The aim of this study is to evaluate the current situation in terms of the treatment used for the management of these fistulae, to analyze its results and to assess its possible predictive factors.

## 2. Materials and Methods

Approval was obtained from the local Clinical Research Ethics Committee from Hospital de la Princesa (Madrid) (09/21-4609).

This is a multicentric national retrospective study of patients operated on for perianal fistula during 2019. The work presented is a sub-analysis of a larger study previously published by this group [3].

### 2.1. Design, Patients and Variables

The Group of Young Colorectal Surgeons of the Spanish Association of Coloproctology (Grupo Joven de la Asociación Española de Coloproctología; GJ-AECP) started this observational retrospective study to analyze surgical preferences in the treatment of the anal fistula (AF) and its results. Neither investigators nor hospitals who participated received financial help for the development of the study. Since the main aim was to analyze its healing rate, a follow-up of at least one year was considered appropriate to obtain more precise data. Follow-up was carried out in external consultations or in patients who had already ended the follow-up by telephone to check if they continued to be asymptomatic or otherwise had to be assessed in consultations again. This telephone consultation consisted of a semi-structured interview in which patients were asked questions in relation to the fistula healing and possible sequelae, mainly fecal incontinence. In such interviews, patients were asked if they were experiencing any kind of perianal or anal swelling, discharge or if they felt any perianal opening or external fistula orifice after the intervention. They were also asked if they have experienced any kind of anal incontinence during at least 6 continuous weeks; any kind of incontinence impairment that was not present preoperatively was documented: gases, liquids or stool and both voluntary and involuntary incontinence. Any time that the patient declared any symptoms that warranted suspicions of the fistula persisting or recurrent and/or continence impairment, those patients were scheduled for a clinical in-person evaluation.

### 2.2. Inclusion and Exclusion Criteria

The inclusion criteria established were 18 years or older, female, diagnosis of trans-sphincteric anterior fistula, elective surgery with curative intention and at least one year of follow-up. The exclusion criteria were doubtful preoperatory diagnosis, non-definitive surgical treatment and patients’ rejection to participate in the study.

Definitions are as follows:

- AF healing: Total absence of perianal discharge, swelling or persistence of a perianal opening.
- AF recurrence: Disappearance of discharge and/or swelling for at least 6 months followed by fistula reappearance.
- AF persistence: The presence of non-interrupted discharge since surgery.
- Preoperatory FI: The loss of control at passing gas or feces during at least 3 months, having started symptoms within 6 months before surgery.
- De novo postoperative FI: The loss of voluntary control in passing gas or feces during at least 8 weeks in the postoperative period, without previous incontinence.

Every continence measurement was made according to the Cleveland Clinic

Incontinence Score (CCIS) [4] and Rome IV criteria [5], as long as its criteria were fulfilled.

The surgical techniques carried out were divided into 3 groups to make comparisons easier: fistulotomy; sphincter-preservation techniques (advancement flap, inter-sphincteric ligation of the fistula tract [LIFT] and fistulotomy and sphincteroplasty); and minimally invasive procedures with the sphincter (Fistula Laser Closure [FILAC], stem cells check, platelet-rich plasma, collagen paste, fibrin glues, plug and video-assisted ablation of fistula tract [VAAFT]).

In addition, we defined preparatory surgery as the surgeries performed before the one that treats the fistula, in general, a loose seton placement and/or perianal persistent collection drainage when necessary.

### 2.3. Main Outcomes

The main outcome is a nationwide analysis of the techniques carried out in this type of fistulae and their healing, persistence and recurrence rates.

Secondary outcomes are the analysis of the risk factors for no healing and to identify the rates of de novo fecal incontinence and its possible risk factors.

### 2.4. Statistical Analysis

Data obtained on each patient has been introduced using pseudonyms in a database and has been analyzed using the program for statistical data Stata 18.0 (StataCorp LLC, College Station, TX, USA).

The qualitative variables have been defined by the number of events and percentages. The quantitative variables that follow normal distribution are described by the average and the standard deviation (SD) and for those that do not follow a normal distribution, by the median and interquartile range.

To know if the variables follow a normal distribution, the Kolmogorov–Smirnov test was employed. For the bivariant analysis of the quantitative variables the T-Student method was applied (in case of having two averages) or the ANNOVA test (more than two averages). To compare qualitative variables that do not follow normal distribution, we used the U-Mann–Whitney test (comparing 2 averages) or Kruskal–Wallis (more than 2 averages). To compare qualitative variables, the χ2 test or Exact Fisher test were utilized depending on appropriateness. Based on the small sample size, the authors decided to analyze the persistent and recurrent fistulas groups together in order to avoid segmentation of the sample and obtain bigger groups of patients for comparisons between them.

In order to assess the risk factors for healing and de novo postoperative incontinence, a multivariate analysis was performed using a logistic regression. Variables statistically (*p* < 0.1) or clinically (OR < 0.67 or OR > 1.5) significant in the univariate analysis were included in the multivariate analysis. This methodology has been previously described (3) and it is used to ensure that in studies with a limited sample size, clinically relevant differences with a relevant impact are not missed due to a lack of power.

The selection of the definitive model was carried out using the forward stepwise method with an inclusion value in the model of *p* < 0.05 and exclusion of *p* > 0.10. *p* < 0.05 considered to indicate statistical significance (2-tailed test). Multicollinearity was not observed in the development of the definitive model.

## 3. Results

**Figure 1:**
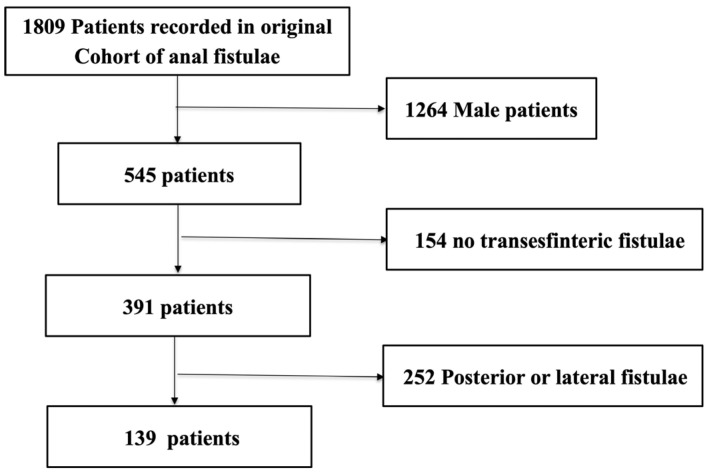
Flowchart of participants in the study.

Initially 1809 patients were included in the database collected from 51 different health centers. Of them, 139 fulfilled inclusion criteria and none the exclusion criteria (Figure 1). Finally, a total of 38 centers were included in this study. The median follow-up was of 18.7 (10.6–28.3) months and, for 80% (111) of patients, were followed by an in-person consultation.

Demographic characteristics and comorbidities are resumed in Table 1.

Of all patients 53.7% had undergone at least one pregnancy, 27 (19.4%) had episiotomy and 13 (9.4%) had undergone instrumentalized labor.

The majority of fistulae were medium height (48%), with 36% low and 16% high. The most frequent technique was fistulotomy and was observed in a total of 29.5% of procedures followed by LIFT with a 22.3% of representation (Table 2).

The global healing rate was 60.4%. The best results were evidenced with fistulotomy (87.8%) followed by sphincter-preservation techniques (51.8%). In Table 3, the healing rate is described according to the height of the fistula and the treatment undergone. The most utilized techniques for high and medium fistulae were sphincter-preserving techniques, with healing rates of 43.3% and 61.5%, respectively.

The multivariate analysis found, as predictive factors of fistula healing, the absence of smoking [OR 3.14 (CI 1.18–8.34), *p* = 0.021] and the fistulotomy procedure [OR 5.97 (CI 1.65–21.53), *p* = 0.006]. The number of previous surgeries is inversely proportional to the healing rate; thus, a low number of preceding surgeries is a protection factor [OR 0.66 (CI 0.46–0.96), *p* = 0.030] (Table 4 and Table 5).

A total of 12.6% of patients presented postoperative de novo fecal incontinence, with a mean CCIS of 3.6 (2–6.8). If we assess the height of the fistula, a higher percentage was observed in those presenting with high fistulae (25%), followed by those with low fistulae (15.5%) and medium height fistulae (9.8%). Table 6 shows the relationship between postoperative de novo incontinence and fistula height and the treatment applied. Up to 25% of postoperative de novo incontinence was noted in low fistulae treated with techniques that were not fistulotomy.

To assess the risk of fecal incontinence, uni- and multivariate analyses were performed, finding obesity as the main risk factor [OR 4.13 (CI 1.29–13.24), *p* = 0.017] (Table 7).

**Figure 2:**
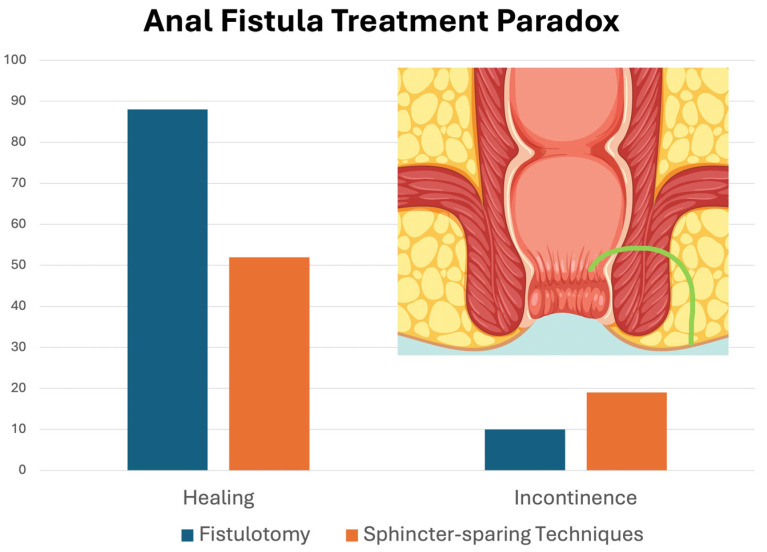
Comparison of healing rate and fecal incontinence after fistulotomy and sphincter-sparing techniques.

Figure 2 compares the main results of healing and fecal incontinence for fistulotomy and sphincter-sparing surgical techniques.

When analyzing two procedures scarcely used (fistulotomy and sphincter repair and cutting seton), we observed that the first one achieved a higher healing rate than the other techniques (91.7% vs. 57.5%, *p* = 0.021) but had a high fecal incontinence incidence (30.0% vs. 11.2%, *p* = 0.085). The opposite was observed for cutting seton, which had a low healing rate (20.0% vs. 61.9%, *p* = 0.060) with low incontinence development (0.0% vs. 13.1%, *p* = 0.387).

## 4. Discussion

The healing rate obtained in this population sample of F-TAAF has been modest, at 57%, with a de novo proportion of fecal incontinence after the different treatments of 12%. A huge variability of treatments was noted: 12 different surgical techniques were performed to resolve this type of fistula.

The most frequently utilized techniques were those classified as sphincter-preserving techniques (LIFT, endorectal flap, fistulotomy with sphincterography). We highlight the non-negligible use of fistulotomy (29.5%).

Even though what exactly motivated the choice of the surgical technique was not assessed in every case, it is logical to think that these interventions, just like minimally invasive procedures, have been selected mostly not for their rate of healing but to prevent impairing fecal incontinence. In this sense, it is striking to check that the de novo incontinence rates in those cases have been the same or even higher than those after fistulotomy when this has been performed.

Hence, the results obtained in this work manifest the enormous complexity of the problem and the lack of promising or contrasting solutions in this field.

It seems contradictory that a significant percentage of F-TAAFs are treated with fistulotomy, even though they are defined as complex and recent guidelines recommend against it [6]. In addition, in that same publication, some questions are posed: what do we consider a complex fistula and what is the heterogeneity in the literature published about it? Yet they classify anterior fistulae in women as complex, with independence from its anatomy, inter-sphincteric or trans-sphincteric. Considering our results, with a good healing rate and low incontinence development, fistulotomy should be seen as the indicated procedure for patients with low fistulas and medium fistulas with strict selection criteria.

Nevertheless the largest percentage of patients underwent sphincter-preserving techniques following the latest guidelines that concluded that advancement flap, fistulotomy and sphincteroplasty or LIFT were safe techniques in these types of patients [6].

If we evaluate the healing rates according to the technique carried out, on one side, we observe that fistulotomy has a 90% chance of success vs. 52.7% in sphincter-saving techniques and 51.7% in minimally invasive procedures, figures that perfectly correlate with previous publications [7]. These results correlate with those published to date, with the healing rates of fistulotomy globally between 83.6% and 93.7% and always superior to those obtained from other procedures [8,9]. On the other hand, techniques such as LIFT, even not established as an elective treatment for F-TAAF, shows healing rates in different randomized clinical trials such as 69.1% [10,11]. In addition, a very recent systematic review has shown that minimally invasive procedures such as plugs or platelet-rich plasma are themselves a risk factor of healing failure [12].

Another remarkable fact is that a median follow-up of over 18 months was achieved, with 80% of the original sample by in-person consultations, and the other 20% via phone telemedicine consultation, what is probably a growing resource for certain types of patients [13]. The extension of follow-up times in these types of patients is quite important as sometimes initial results are transitory, and definitive results take a little longer time to stablish [14].

To date, no paper uniquely assessing F-TAAF has been published. Furthermore, we can just extrapolate results from studies that analyze healing rates in complex fistulae. For instance, in the nationwide study recently published by the GJ-AECP [3], fistulotomy in complex fistulae showed a healing rate of 79.5% compared with 58% in sphincter-sparing techniques and 38% in minimally invasive procedures. Therefore, we could say that F-TAAF might benefit from the first technique with a certain warrant.

It is a notorious fact that the reduced percentage of patients who obtained healing did so through sphincter-sparing techniques even in F-TAAF medium or low fistulae and that healing was obtained in a smaller percentage in those treated with minimally invasive techniques. According to the literature, sphincter-sparing mini-invasive techniques such as FiLAC are an optimal indication for high trans-sphincteric fistula with healing rates of 60% and 76.9% in patients with Crohn’s disease, results that are better that those published in our study, where the target is only F-TAAF [15,16]. This should make us reconsider if a better selection of patients could persuade the encouragement of fistulotomy to a greater number of them, putting to one side sphincter-sparing and minimally invasive techniques.

On the one hand, in the uni- and multivariate analysis, the main risk factors for non-healing have been the performance of a technique that is not fistulotomy and the need of several previous surgeries. This last point has also been demonstrated in a previous study about complex fistula treatment with rates of failure of healing of about 18.1% [17]. All of this runs in favor of reconsidering fistulotomy as a technique to take into account in this type of patient, reducing the number of previous surgeries and reducing the risk of non-healing. On the other hand, another risk factor for non-healing and fistula recurrence is well-known active smoking [18,19]; so, it is essential to insist on this aspect to patients in order to obtain better healing results, although the literature is not unanimous in every recurrence risk factors for anal fistula [20].

If we assess results in terms of de novo incontinence, we will observe that sphincter-saving techniques are not exempted from incontinence. In fact, this rate was higher in this group than in fistulotomy (19.2% vs. 10.3%). This incontinence rate was even higher in patients with low fistula who underwent these techniques (25%), despite the number of patients in this group being low. This fact is especially notorious since these fistulae, according to results, could be operated on with fistulotomy with lesser risk of de novo incontinence. Concerning risk factors identified in this study for the appearance of de novo incontinence, none has been found to be related to the operating techniques. Obesity is the only risk factor, already known to be a preoperative incontinence risk factor [21]. However, this observation in the present study could be refuted by several other articles that have shown opposite results [22,23].

It is clearly known that fecal incontinence is a multifactorial issue [24]. What seems straight is that in these cases the appearance of incontinence has no exclusive relation with the section of the sphincters during fistulotomy. Furthermore, an important fecal incontinence rate has been described even with techniques that a priori respect sphincters. Once again, one of the incontinence risk factors has been the number of previous procedures [3,17]. Thus, the use of sphincter-preserving techniques that are not associated with higher healing, since they usually require multiple surgeries, could be adding a similar or even greater risk to that generated by the section of the sphincter during fistulotomy. Therefore, fistulotomy should be seen as the indicated procedure for patients with low fistulas and medium fistulas with strict selection criteria. A possible alternative that has been published with good results in complex anal fistula, is fistulotomy with immediate sphincter repair, although it is also related to non-depictable de novo incontinence rates of close to 10% [25].

The pathophysiological mechanism by which sphincter-sparing techniques worsen fecal incontinence is difficult to understand. As it has been previously said, the need of multiple procedures with multiple anal dilatations could contribute to the development of this complication [26]. Another possible explanation would be the damage, limited but real, of the anal sphincter in certain sphincter-sparing techniques, such as laser or radiofrequency fistula treatment, which may affect the anal canal with scarring and impairing anodermal sensitivity [27]. Finally, another possible reason would be the damage in anal sphincter function due to a persistent anal fistula not cured by one of these procedures. The existence of persistent suppuration and inflammation could also be a reason for this de novo fecal incontinence. All these hypotheses are speculated and should be further assessed in well-designed studies aimed at analyzing sphincter-sparing techniques.

Definitively, just as it occurred in the original paper that lead to the present sub-analysis, a message that the authors would like to convey is the fact that the best opportunity to reach the best solution for patients is the moment of the initial evaluation of the primary fistula since it seems that successive manipulations, even if they are careful with the sphincters, could imply a risk both for healing and for incontinence of the patients. Consequently, it is mandatory to perform judicious exercises on the use of the different surgical techniques, balancing the opportunities for healing and the risk of incontinence.

There are two fundamental limitations to this study. The main one is that it is retrospective, which leads to the second important limitation, namely that it is not possible to know some of the details of why each surgical team opted for the surgical technique used in each case. Therefore, it is impossible to know whether the chosen technique is the one that has always been considered best for patients or whether decisions may have been influenced by training deficiencies or lack of knowledge of other techniques. Considering also that there have been few cases despite the high participation of 39 centers, all these arguments may be of particular interest when it comes to decisions and results obtained in centers that are very different from each other.

Despite these limitations the present study has important strengths that must be highlighted. These are the fact that it is one of the first times that a specific analysis of fistulae in female patients (F-TAAF) is performed, is a representation of a high volume of centers, is a meticulous statistical analysis and reflects a very pertinent and daily problem. In spite of this, the need to develop prospective studies with a superior design to assess the concrete problem in this type of fistulae is obvious since it is not fulfilled yet.

Being conscious about our design limitations in the present retrospective work, it is evident that we cannot recommend fistulotomy as a first line treatment, and we consider that it should be selected cautiously, in general, and tremendously selectively in cases of F-TAAF. That said, the results obtained with the rest of techniques have been much worse in terms of healing and, paradoxically, have not been better concerning continence. In fact, there is a group in which the results in FI have been worse than fistulotomy, and this group was one of the better-balanced groups after segmentation of the sample. This observation leads to the impossibility of arguing in favor of recommending none of the alternative treatments in this context.

## 5. Conclusions

Assessing these results, we can conclude that the treatment of F-TAAF is a challenge with a total of 12 different surgical procedures and generally discrete results with regards to healing. Nevertheless, and despite its potential risks, the healing rate of patients treated with fistulotomy is quite high without a higher rate of incontinence, compared with the rest of the techniques. For all that, we think that fistulotomy should be seen as the indicated procedure for patients with low fistulas and medium fistulas with strict selection criteria.

In view of the present analysis, it has been shown that it is a very challenging condition, with enormous variability in terms of surgical techniques employed and with overall results that could be considered mediocre. Maintaining very strict selection criteria and with favorable anatomical conditions, fistulotomy appears to have the best cure rates without a significantly higher impact on continence. Cases with anatomically unfavorable fistulae are a major clinical problem as there is no clear standard of care and the different existing alternatives present poor results.

## Figures and Tables

**Table 1:** Demographic features of the patients included. Data are expressed as number of patients and percentage.

		n = 139
Age (years) *		45.8 (43.6–47.9)
Duration of symptoms before surgery (months) *		21.2 (17.7–24.6)
Obesity (BMI > 30)		46 (35.3%)
Diabetes		11 (7.9%)
Current smoker		33 (24.6%)
Inflammatory bowel disease		14 (10.1%)
Previous anal surgery		71 (51%)
Previous AF surgery		41 (29.5%)
Patients with 2 or more anal fistula surgeries		13 (9.3%)
Previous perianal abscess		86 (61.9%)
Previous delivery (in women)		58 (53.7%)
Preparatory surgeries before the definitive procedure	None	38 (27.34%)
	1	55 (39.57%)
	2	31 (22.3%)
	3 or more	15 (10.79%)
Preoperative fecal incontinence		6 (4.3%)
Cleveland Clinical Incontinence Score in patients with preoperative FI *		3.8 (2.2–6.4)

* Mean and 95% CI values.

**Table 2:** Anatomical fistula classification and surgical procedures of the patients included.

		n = 139
Trans-sphincteric fistula classification	Low	50 (36%)
	Medium	67 (48%)
	High	22 (16%)
Multiple tracts		11 (7.9%)
Associated abscess		14 (10%)
Cryptoglandular origin		120 (83.6%)
Surgical procedure	Fistulotomy	41 (29.5%)
	LIFT	31 (22.3%)
	Platelet-rich plasma	14 (10%)
	Advancement flap	13 (9.3%)
	Fistulotomy + sphincter repair	12 (8.6%)
	Drain seton	8 (5.7%)
	Fibrin sealant	8 (5.8%)
	FiLaC	5 (3.6%)
	Cutting seton	5 (3.6%)
	Stem cells	1 (0.7%)
	Anal plug	1 (0.7%)

Data are expressed as the number and percentages of patients. LIFT: ligation of the inter-sphincteric fistula tract; FiLaC: fistula laser closure.

**Table 3:** Postoperative healing stratified for fistula type and surgical interventions: fistulotomy vs. sphincter-sparing surgical techniques vs. sphincter-sparing minimally invasive procedures.

		Fistulotomy	Sphincter-Sparing Surgical Techniques	Sphincter-Sparing Minimally Invasive Procedures	*p*
Low fistula	Healing (n = 36, 78.3%)	26 (89.7%)	8 (61.5%)	2 (50%)	0.04
	Non-healing (n = 10, 21.7%)	3 (10.3%)	5 (38.5%)	2 (50%)	
Medium fistula	Healing n = 34, 54.0%)	10 (83.3%)	13 (43.3%)	11 (52.4%)	0.06
	Non-healing (n = 29, 46.0%)	2 (16.7%)	17 (56.7%)	10 (47.6%)	
High fistula	Healing (n = 10, 58.8%)	--	8 (61.5%)	2 (50%)	0.68
	Non-healing (n = 7, 41.2%)	--	5 (38.5%)	2 (50%)	

Patients who underwent a draining seton or cutting seton could not be included in this table as they did not fit in any of the surgical intervention categories (n = 126).

**Table 4:** Factors associated with anal fistula healing. Data are expressed as number and percentage of patients.

		Healing (84, 60.4%)	Non-Healing (55, 39.6%)	OR (95%-CI)	*p*
Age (years) *		46.2 (43.3–48.8)	45 (41.3–48.7)	-	0.5
Duration of symptoms (months) *		19.3(14.8–23.8)	24 (18.4–29.6)	-	0.05
Obesity		22 (28.2%)	24 (46.1%)	0.45 (0.22–0.95)	0.04
Diabetes		5 (5.95%)	6 (11%)	0.5 (0.1–1.6)	0.2
Current smoker		14 (17.3%)	19 (35.8%)	0.37 (0.17–0.83)	0.01
Previous anal surgery		38 (45.2%)	33 (60%)	0.5 (0.2–1.09)	0.08
Previous anal fistula surgery		22 (26.2%)	19 (34.5%)	0.6 (0.3–1.2)	0.2
Previous perianal abscess		48(57.1%)	38(69.1%)	0.5 (0.2–1.2)	0.15
Previous delivery		37 (52.1%)	21 (56.8%)	0.8 (0.4–1.8)	0.65
Preparatory surgeries before the definitive procedure	None	27 (32.1%)	11 (20%)	3.28 (2.32–4.64)	0.00
	1	34 (40.4%)	21 (38.2%)	2.19 (1.56–3.08)	<0.01
	2	15 (17.9%)	16 (29.9%)	1.53 (1.03–2.27)	0.02
	3 or more	8 (9.5%)	7 (12.7%)	--	
Preoperative fecal incontinence		2 (2.3%)	4 (7.2%)	0.3 (0–1.5)	0.1
Multiple tracts		5 (6%)	6 (10%)	0.5 (0.1–1.6)	0.2
Associated abscess		5 (5.9%)	9 (16.3%)	0.3 (0.1–0.9)	0.046
Anal Fistula Origin	Cryptoglandular origin	78 (92.9%)	47 (85.4%)	0.5 (0.1–1.6)	0.2
	Inflammatory bowel disease	6 (7.1%)	8 (14.5%)		
Surgical procedure	Fistulotomy	36 (45%)	5 (10.8%)	8.6 (6.31–11.98)	0.03
	Sphincter-sparing surgical techniques	29 (36.2%)	27 (58.7%)	2.21 (1.55–3.14)	<0.01
	Sphincter-sparing mini-invasive procedures	15 (18.7%)	14 (30.4%)	--	

* Mean and 95% CI values. OR: Odds Ratio; 95%-CI: 95% confident interval.

**Table 5:** Multivariate analysis for anal fistula healing.

Variables		OR	95%-CI	*p*
Surgical procedure	Sphincter-sparing mini-invasive procedures	Reference	-	-
	Fistulotomy	5.9	(1.7–21.6)	0.006
	Sphincter-sparing surgical techniques	0.8	(0.3–2.1)	0.608
Current smoker		3.1	(1.2–8.3)	0.021
Number of preparatoy surgeries		0.7	(0.5–0.96)	0.03

**Table 6:** New-onset postoperative incontinence stratified for fistula type and surgical interventions: fistulotomy vs. sphincter-sparing surgical techniques vs. sphincter-sparing mini-invasive procedures.

		Fistulotomy	Sphincter-Sparing Surgical Techniques	Sphincter-Sparing Mini-Invasive Procedures	*p*
Low fistula	FI (n = 7, 15.6%)	3 (10.3%)	3 (25.0%)	1 (25.0%)	0.430
	No FI (n = 38, 84.4%)	26 (89.7%)	9 (75.0%)	3 (75.0%)	
Medium fistula	FI (n = 6, 9.8%)	1 (9.1%)	3 (10.3%)	2 (9.5%)	0.991
	No FI (n = 55, 90.5%)	10 (90.9%)	26 (89.7%)	19 (90.5%)	
High fistula	FI (n = 4, 25.0%)	--	4 (33.3%)	0	0.182
	No FI (n = 12, 75.0%)	--	8 (66.7%)	4 (100%)	

FI: fecal incontinence. Patients who underwent draining or cutting seton were not included in this table as they did not fit in any of the surgical categories. Neither were the patients with preoperative fecal incontinence included (n = 122).

**Table 7:** Factors associated with postoperative anal incontinence.

Variables	Postoperative FI	No Postoperative FI	OR (95%-CI)	*p*
Age (years) *	47.6 (42.4–52.8)	45.4 (43–47.8)	-	0.6
Obesity	9 (64.2%)	34 (30.6%)	4.13 (1.34–12.65)	0.02
Diabetes	2(11.7%)	9 (7.6%)	1.6 (0–7.39)	0.55
Current smoker	4 (28.5%)	27 (23.2%)	1.3 (0.4–4.3)	0.6
Inflammatory bowel disease	1 (5.8%)	13 (11%)	0.5 (0–3.2)	0.51
Previous anal surgery	9 (53%)	59 (50%)	1.13 (0.42–3.03)	1
Previous anal fistula surgery	8 (47%)	30 (25.4%)	3 (0.7–12)	0.1
Time of symptoms *	20.9 (17.1–24.7)	20.6 (10.5–30.7)	-	0.06
Previous perianal abscess	4 (23.5%)	79 (67.0%)	0.2 (0.05–0.5)	<0.01
Preparatory surgeries before definitive procedure				
None	7 (41.2%)	31 (26.2%)	-	-
1	6 (35.2%)	47 (39.8%)	0.57 (0.17–1.84)	0.612
2	3 (7.6%)	27 (22.8%)	0.49 (0.12–2.09)	
3 or more	1 (5.8%)	13 (11%)	0.34 (0.04–3.05)	
Anal fistula characteristics				
Multiple tracts	2 (11.7%)	8 (6.7%)	1.8 (0–8.5)	0.4
Associated abscess	0	13 (11%)	0 (0–1.8)	0.1
Cryptoglandular origin	15 (93.7%)	101 (87.8%)	0.48 (0.05–3.9)	0.5
Surgical procedure				
Fistulotomy	4 (23.5%)	36 (34.2%)	---	0.386
Sphincter-sparing surgery	10 (58.8%)	43 (41%)	2.09 (0.60–4.12)	
Sphincter-sparing mini-invasive procedures	3 (17.6%)	26 (24.7%)	1.04 (0.21–3.68)	

* Mean and 95% CI values. OR: Odds Ratio; 95%-CI: 95% confident interval.

## Data Availability

The data will be accessible to researchers who make reasonable requests to the authors.

## Abbreviations

The following abbreviations are used in this manuscript:

AF	anal fistula
F-TAAF	female trans-sphincteric anterior anal fistula
FI	fecal incontinence
GJ-AECP	Group of Young Colorectal Surgeons of the Spanish Association of Coloproctology (Grupo Joven de la Asociación Española de Coloproctología)
CCIS	Cleveland Clinic Incontinence Score
LIFT	inter-sphincteric ligation of the fistula tract
FiLaC	fistula laser closure
VAAFT	video-assisted ablation of fistula tract
SD	standard deviation
